# Understanding How Social Media Use Relates to Turnover Intention Among Chinese Civil Servants: A Resource Perspective

**DOI:** 10.3390/bs15101331

**Published:** 2025-09-28

**Authors:** Min Hua, Yuanjie Bao

**Affiliations:** School of Public Administration and Policy, Renmin University of China, Beijing 100872, China; minhua@ruc.edu.cn

**Keywords:** excessive social media use at work, social media use for work during non-work hours, social media exhaustion, turnover intention, resilience, conservation of resources theory

## Abstract

The rise in social media has blurred work–life boundaries, and concerns have been raised about its impact on employee well-being. This study examines how excessive social media use at work (ESMU) and social media use for work during non-work hours (SMUNW) affect turnover intention. Social media exhaustion is tested as a mediator, and resilience is tested as a moderator. Survey data were collected from 453 civil servants in Shandong Province, China. Hierarchical regression and the PROCESS MARCO were used for analysis. The results indicate that ESMU (*β* = 0.42, *p* < 0.001) and SMUNW (*β* = 0.14, *p* < 0.01) both significantly increase turnover intention. Social media exhaustion mediates these relationships, while resilience reduces their negative impact. Our findings contribute to technostress research by clarifying how digital demands influence public employees. For managers and organizations, the results highlight the need to set boundaries for work-related social media use; monitor employees’ digital exhaustion; and foster resilience through recruitment, training, and organizational support.

## 1. Introduction

With the rapid development of information and communication technology (ICT), social media has become widely accepted and extensively used (Correa et al., 2010). It provides a platform for sharing opinions, insights, and experiences, while also enabling timely communication and reducing delays in information transmission (L. Shi et al., 2022). However, just as ICT itself is a double-edged sword (C. Zhang et al., 2022), the use of social media also presents both opportunities and risks (Leong et al., 2019). While it adds convenience to people’s work and life, it has also been associated with a range of impairments (Ali-Hassan et al., 2015; Blackwell et al., 2017; Hawi & Samaha, 2017; Su et al., 2020).

Recognizing these negative consequences, scholars have examined the detrimental effects of social media use in the workplace. For example, studies have shown that excessive social media use at work (ESMU) reduces individual work performance through social media exhaustion (Yu et al., 2018; Cao & Yu, 2019) and increases procrastination and interruptions, thereby lowering work engagement (B. Wang et al., 2023). Other research has highlighted the impact of social media use for work during non-work hours (SMUNW), which intensifies work–family conflict and reduces engagement (F. Wang & Li, 2023). Collectively, these findings indicate that both ESMU and SMUNW can undermine employee outcomes. However, prior studies have typically examined these two forms of social media use in isolation, and no research has simultaneously investigated the influence of both within a unified framework.

In addition, most existing studies focus on private sector employees (Ali-Hassan et al., 2015; Kalra et al., 2023; Kanwal et al., 2023; Ma et al., 2023), leaving the public sector largely overlooked. This omission is significant, as civil servants often face higher pressure to remain available to citizens both during and after working hours (F. Wang & Li, 2023). In China, the integration of digital governance into public administration has further expanded the reliance on social media for public service delivery, policy communication, and citizen engagement. Civil servants are frequently required to respond to inquiries, coordinate administrative tasks, and handle citizen demands through social media platforms, creating an expectation of “24 h connectivity”. While this enhances efficiency and transparency, it also leads to negative consequences such as heightened job stress, emotional exhaustion, and work–family conflict. The dual pressures of public accountability and the demand for continuous responsiveness make civil servants particularly vulnerable to the risks associated with social media use. Combined with the high turnover rate in the Chinese civil service, these features underscore the necessity of examining the impacts of social media use in this group.

Against this backdrop, this study developed and tested a model based on Conservation of Resources (COR) theory and positive psychology. COR theory posits that individuals strive to acquire, retain, and protect valued resources and that resource depletion exerts stronger effects than resource acquisition. This perspective helps explain how ESMU and SMUNW may drain psychological resources and foster exhaustion, which in turn increases turnover intention. Positive psychology highlights the role of personal strengths and positive resources, such as resilience, in mitigating stress and promoting well-being. Accordingly, resilience is introduced as a moderator that buffers the negative effects of resource depletion caused by social media use.

This study contributes to the literature in four ways. Firstly, it is the first to integrate ESMU and SMUNW into the same research framework, thereby providing a more comprehensive understanding of work-related social media use. Secondly, it clarifies the mediating role of social media exhaustion in the relationship between social media use and turnover intention, responding to calls for greater attention to the mechanisms underlying this effect (Cho et al., 2019; Tang et al., 2020; Wu et al., 2021). Thirdly, drawing on positive psychology, it explores how resilience mitigates the negative impacts of social media use, thereby enriching our understanding of boundary conditions and offering practical insights for managers. Fourthly, by focusing on Chinese civil servants, this study extends technostress research to an underexplored group. In highlighting the unique digital pressures and accountability demands faced by civil servants, it not only advances theoretical understanding but also offers actionable implications for managing employee well-being and retention in the context of digital governance. Collectively, these contributions advance theory and provide meaningful guidance for managing the challenges of social media use in the public sector.

## 2. Theoretical Background: Conservation of Resources Theory (COR)

Hobfoll (1989) proposed Conservation of Resources Theory (COR) in order to explain individual behavior under stressful situations. With more than thirty years of development and application, COR theory has become a mainstream explanatory theory in the field of technostress and in the related social media use literature (Halbesleben & Bowler, 2007). The basic assumption of COR theory is that individuals have the motivation to preserve, protect, and acquire resources, and, when faced with potential or actual resource losses, individuals will experience anxiety and stress (Hobfoll, 1989; Hobfoll et al., 2018). In order to cope with pressure, individuals will adopt two methods. One is to use existing resources to acquire new resources, thereby reducing actual and potential resource losses. The other method is to actively take measures to build and maintain existing resources to cope with environmental pressure.

Hobfoll et al. (2018) further proposed that the preservation and acquisition of resources follow four basic principles: (1) Resource loss is disproportionately more salient than resource gain. Compared with resource gain, resource loss affects individuals more quickly, lasts longer, and is therefore more important to the individual. Therefore, the actual or potential loss of resources caused by work demands will lead to tension and stress reactions, including burnout (Hobfoll & Freedy, 2017; Shirom, 1989), reduced well-being (Wright & Hobfoll, 2004), depression (Bolger et al., 1989), and other negative physical, psychological, and work outcomes. (2) Individuals need to protect existing resources and acquire new resources through resource investment. Halbesleben and Bowler (2007) pointed out that in the case of resource loss, individuals will increase resource investment. (3) Paradoxically, in the case of resource loss, it is particularly important to acquire new resources. When individuals facing the depletion of resources receive an injection or increase in resources, the effect of relieving tension and pressure is more obvious. (4) The fourth principle of COR theory is resource desperation; when an individual’s resources are depleted, the individual will enter defensive mode to protect themselves, are often aggressive, and may become irrational. As with other aspects of COR theory, this may be a built-in evolutionary strategy and may be defensive (i.e., to conserve resources) or exploratory (i.e., to find alternative survival or adaptation strategies).

This study used COR theory as a theoretical basis to explore the effects and mechanisms of social media use during work and non-work hours. For public officials, the institutional expectation of accountability, transparency, and continuous responsiveness places them in a work environment where the boundaries between professional and personal life are blurred. In this context, ESMU and SMUNW function as persistent stressors that accelerate resource loss by consuming time, energy, and emotional capacity. The disproportionate impact of such losses, as emphasized in COR theory, is particularly salient for civil servants, who often have fewer opportunities for recovery compared with private sector employees.

In this study, ESMU and SMUNW are two stressors that lead to exhaustion in employees, which in turn causes employees to develop turnover intention. Psychological resilience is an important resource (Bardoel & Drago, 2021) and represents an individual’s ability to recover, regroup, adjust, and even thrive after encountering misfortune, change, or adversity (Garcia-Dia et al., 2013). In the context of civil service, resilience operates as a critical personal resource that helps officials cope with the continuous demands of digital governance and the erosion of psychological resources caused by social media use. By linking the distinctive pressures of public officials with the principles of COR theory, this study highlights both the mechanisms of resource depletion and the protective function of resilience in a high-demand, accountability-driven environment.

## 3. Hypothesis Development

### 3.1. ESMU, SMUNW, and Turnover Intention

Public sectors in many countries are facing the problem of high employee turnover rates, such as Pakistan (Asif et al., 2023), Malaysia (Roslan et al., 2014), South Korea (Kim, 2018), and the Sultan of Oman (Al Balushi et al., 2022). This phenomenon poses a serious challenge to the public sector management. A high turnover rate means that organizations need to constantly select and train new employees, greatly increasing the economic costs to the public sector (Caillier, 2016). In addition, rapid personnel turnover is not conducive to the development of longer cyclical work in the public sector. Turnover intention is defined as ‘the estimated probability that one will leave the organization at some future time’ (Hughes et al., 2010, p. 353) and is an important prerequisite to turnover behavior (Sun & Wang, 2017). Research in the public sector has shown that work stress is an important antecedent of turnover intention among nurses (Samad et al., 2021), healthcare personnel (Herttalampi & Feldt, 2023), and teachers (Husain et al., 2016).

Although the original intention of social media was to facilitate people’s work and life, the excessive use of social media has caused people stress and produced many adverse consequences. For example, C. Shi et al.’s (2020) research showed that the social media overload faced by college students leads to experienced stress. Whelan et al. (2020) proved the strong correlation between social media overload and fatigue. Similarly, a large-scale survey study by Lutz et al. (2014) showed that social media overload causes stress and tension in users.

According to the COR theory, individuals feel nervous and stressed when faced with potential or actual resource losses (Hobfoll, 1989; Hobfoll et al., 2018). In the face of this pressure and tension, people might generate turnover intention to protect and detach themselves from a negative environment. According to the COR theory (Halbesleben & Bowler, 2007; Hobfoll, 1989; Hobfoll et al., 2018), it can be deduced that both ESMU and SMUNW would lead to significant resource loss among civil servants, thus leading them to experience negative effects such as anxiety, pressure, and stress. Therefore, civil servants might develop turnover intention to protect their limited resources, prevent future resources loss, and detach themselves from a stressful situation that is detrimental to their valued resources.

Empirical evidence suggests that both ESMU and SMUNW can lead to turnover intention. Specifically, studies have indicated that ESMU can cause turnover intention (Bizzi, 2018; Cho et al., 2019; Choi, 2018). Similarly, SMUNW is inextricably linked to an increase in turnover intention (Tang et al., 2020). Based on theoretical reasoning and empirical evidence, it is hypothesized that

**H1a.**  *ESMU is positively associated with turnover intention*.

**H1b.**  *SMUNW is positively associated with turnover intention*.

### 3.2. The Mediating Role of Social Media Exhaustion

Exhaustion refers to the depletion of mental resources caused by prolonged exposure to high-demand environments (Schaufeli et al., 2009). Social media exhaustion describes an individual’s sense of exhaustion caused by social media use (Maier et al., 2015). Social media exhaustion refers to an individual’s aversion and unconscious psychological reaction to stress in a social media environment and social media-related overload caused by the excessive use of social media. When individuals are exposed to an overloaded environment related to social media, they are likely to experience social media exhaustion (Yu et al., 2018).

According to COR theory, when faced with the potential loss of resources, individuals may feel tense and stressed, and the accumulation of stress and tension leads to exhaustion (Hobfoll, 1989; Hobfoll et al., 2018). This study posited that the positive impact of ESMU on social media exhaustion can be explained through two aspects. On the one hand, the excessive use of social media at work requires employees to always pay attention to information from leaders and colleagues on social media and respond in a timely manner, which takes up employees’ normal working time. This means that employees need to complete their work in a shorter span of time. Having to complete work tasks within a time limit will undoubtedly increase the pressure on employees. On the other hand, ESMU disrupts the normal work rhythm of employees, forcing their working time to be cut into fragmented periods. This will lead to employees being unable to concentrate and carry out their work with engagement and involvement. Therefore, work efficiency will decrease, leading to tension and stress. For these reasons, we conjectured that ESMU would lead to social media exhaustion, and there is initial empirical evidence supporting this relationship (Fu et al., 2020).

Similarly, it can be inferred that SMUNW will also result in social media exhaustion. Firstly, when employees face SMUNW, their individual resources such as energy, physical strength, and emotions can be drained. SMUNW causes resource depletion, because employees are not able to recover from work during non-work hours. In this regard, the normal recovery of resources during non-work hours is hindered. Secondly, SMUNW forces employees to handle work-related tasks during non-work hours, taking up the time that employees would spend on family affairs and easily triggering work–family conflicts, which represent one of the antecedents to emotional exhaustion (Glaser & Hecht, 2013). Therefore, it can be deduced that there is a positive correlation between SMUNW and social media exhaustion.

When social media exhaustion is high, employees are more likely to have turnover intentions. This is because COR theory states that when resources face depletion, individuals will enter defense mode to protect themselves. Resignation is one of the defensive modes adopted to avoid the continued loss of resources. Thus, it is natural for individuals to develop turnover intentions when facing social media exhaustion induced by ESMU and SMUNW. Empirical studies have also confirmed the positive impact of exhaustion on turnover intention (Saleh et al., 2023; Tang et al., 2020). Based on theoretical reasoning and empirical evidence, it is hypothesized that

**H2a.**  *Social media exhaustion mediates the relationship between ESMU and turnover intention*.

**H2b.**  *Social media exhaustion mediates the relationship between SMUNW and turnover intention*.

### 3.3. The Moderating Role of Resilience

Psychological resilience is defined as a person’s ability to recover, re-bounce, adjust, and even thrive after misfortune, change, or adversity (Garcia-Dia et al., 2013). Recently, the stress literature has highlighted resilience as an important boundary condition when exploring the influence of stress (Cole et al., 2015; García-Izquierdo et al., 2018; Palm-Fischbacher & Ehlert, 2014; Stanley et al., 2021). However, in the research field of social media stress, few scholars have used resilience as a moderating variable to explore its buffering effect. Resilience, as a positive trait, can also be regarded as a type of personal resource that individuals can utilize when necessary to achieve their desired goals.

We examined how resilience as a type of personal resource would mitigate the influences of both ESMU and SMUNW on social media exhaustion. According to the COR theory, when faced with resource loss, individuals will increase resource investment to cope with the current unfavorable situation (Halbesleben & Bowler, 2007). ESMU requires individuals to pay attention to social media trends at all times while working, and to respond and process information posted on social media by leaders and colleagues in a timely manner. This causes a loss of physical, energy, emotional, and other resources in individuals, and they will choose to increase their resource investment in response. Resilience is an important resource possessed by individuals. When an individual’s level of resilience is high, they can adjust to and recover from stressful situations more quickly. In this case, the impact of ESMU on social media exhaustion is weaker.

Similarly, SMUNW forces individuals to use social media to deal with work-related matters during off-duty hours. In addition to costing the individual physical, energy, emotional, and other resources, this also consumes their leisure time and resources. COR theory states that individuals will invest resources to make up for a loss of resources that has occurred (Halbesleben & Bowler, 2007). Consequently, individuals who process a high level of resilience will have more resources when dealing with the stressful situation created by SMUNW. Through the supplementation of resources through resilience, the impact of SMUNW can be alleviated, leading to individuals feeling less social media exhaustion. It is therefore hypothesized that

**H3a.**  *Resilience moderates the relationship between ESMU and social media exhaustion, such that the relationship is weaker when resilience is high.*

**H3b.**  *Resilience moderates the relationship between SMUNW and social media exhaustion, such that the relationship is weaker when resilience is high.*

The above hypotheses indicate that there are indirect relationships between the two independent variables, ESMU and SMUNW, and the dependent variable of turnover intention, and the relationship between the two independent variables and the mediating variable social media exhaustion is moderated by resilience. Therefore, we speculate that resilience moderates the two indirect relationships between ESMU and SMUNW and turnover intention (Preacher et al., 2007). When employees’ resilience is higher, these indirect effects will be mitigated. It is hypothesized that

**H4a.**  *Resilience moderates the indirect effect of ESMU on turnover intention through social media exhaustion; the indirect effect is weaker when resilience is high*.

**H4b.**  *Resilience moderates the indirect effect of SMUNW on turnover intention through social media exhaustion; the indirect effect is weaker when resilience is high.*

**Figure 1:**
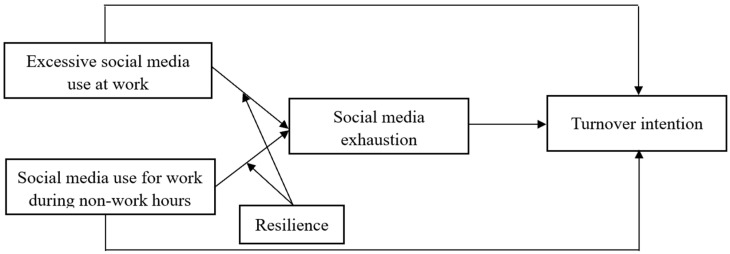
Hypothesized model.

All proposed hypotheses are depicted in Figure 1.

## 4. Methods

### 4.1. Sample and Procedure

This study collected survey data from civil servants working in Shandong Province, China. Participants were informed that participation was voluntary, confidential, and anonymous. In November 2023, we distributed 530 invitation emails to civil servants, inviting them to complete an online questionnaire. By December 2023, a total of 504 responses had been received. Following data quality checks, 51 questionnaires were excluded because participants failed the attention check item (“This question has no actual meaning, please select option A”), resulting in 453 valid responses for the final analysis. The valid response rate was 85.5%. Prior to the formal survey, a pilot test was run to assess the clarity and reliability of the measurement scales, and the results indicated satisfactory performance.

Among the 453 valid participants, 369 were female (81%) and 84 were male (19%). This distribution reflects the actual workforce composition of the surveyed departments rather than sampling bias and should not affect the validity of the analyses or the robustness of the results. The age distribution was as follows: 21–29 years (35%), 30–39 years (37%), 40–49 years (16%), and 50–60 years (12%). Regarding tenure, 136 participants (30%) had worked for 1–5 years, 118 (26%) for 6–10 years, 66 (15%) for 11–15 years, 39 (9%) for 16–20 years, 52 (11%) for 21–30 years, and 42 (9%) for 31–45 years. In terms of marital status, 337 participants (74%) were married and 116 (26%) were unmarried. Table 1 shows the summary statistics for the demographic information of the respondents.

### 4.2. Measures

All items in this study were measured using a 6-point Likert-type scale (1 = strongly disagree, 6 = strongly agree), except for the control variables (e.g., gender, age, tenure, and marital status), which were measured using their appropriate formats. To ensure validity and reliability, we employed measurement scales that have been previously applied in Chinese samples and demonstrated satisfactory psychometric properties. The details of each variable are given in the following subsections, and all measurement items are provided in Table A1 in Appendix A.

#### 4.2.1. Excessive Social Media Use at Work

A three-item scale (Yu et al., 2018) was used to measure ESMU. An example item was “I think the amount of time I spend using social media at work is excessive”. The Cronbach’s α was 0.918.

#### 4.2.2. Social Media Use for Work During Non-Work Hours

SMUNW was measured with the four-item scale adapted by F. Wang and Li (2023), which was originally developed by Derks et al. (2016) and Leftheriotis and Giannakos (2014). One example item was “I often use social media to obtain work related information and knowledge during non-work hours”. The Cronbach’s α was 0.904.

#### 4.2.3. Social Media Exhaustion

Four items were used to measure social media exhaustion (Yu et al., 2018). An example item was “Working all day with social media is a strain for me”. The Cronbach’s α was 0.976.

#### 4.2.4. Resilience

Resilience was measured with a psychological capital questionnaire developed by Luthans et al. (2007). Resilience is an aspect of psychological capital and was measured using six items. A sample item was “I usually take stressful things at work in stride”. The Cronbach’s α was 0.794.

#### 4.2.5. Turnover Intention

A three-item turnover intention scale (Liang, 1999) was used to measure turnover intention. A sample item was “I often think about leaving this organization”. The Cronbach’s α was 0.939.

#### 4.2.6. Control Variables

The respondents’ gender (0 = female, 1 = male), age (in years), tenure (in years), and marital status (1 = unmarried, 2 = married) were included as control variables, as they might be related to turnover intention (Ha et al., 2024; Ju & Li, 2019).

### 4.3. Data Analysis Methods

This study employed multiple statistical software packages and analytical techniques to ensure the robustness of the results. Firstly, confirmatory factor analysis (CFA) was conducted to test the convergent and discriminant validity of the variables, and AMOS 29.0 was used. Secondly, the potential issue of common method bias was examined using Harman’s single-factor test (Harman, 1976) using SPSS 26.0.

For hypothesis testing, this study used hierarchical regression analysis to examine the direct effects (H1a and H1b) and mediating effects (H2a and H2b). To test the moderating effects (H3a and H3b), following the recommendations of Aiken et al. (1991), the variables were mean-centered before the construction of the product term, and a simple slope analysis was conducted. Finally, to test the moderated mediation effects (H4a and H4b), this study employed the PROCESS MARCO developed by Hayes (2017) to examine the indirect effect and its significance under different values of the moderator variable.

### 4.4. Common Method Bias Test

The issue of common method bias should be addressed since all measures were obtained from a single source with self-reported measures. In order to respond to this problem, we checked whether common method bias poses a serious problem using Harman’s single-factor test (Harman, 1976). Exploratory factor analysis (EFA) on all items revealed five factors, which explained 79.453% of the variance. The first main factor explained 36.659% of the variance. Since this is below the recommended threshold value (<40%) and the single-factor result did not explain the majority of the variance (Saris & Gallhofer, 2014), it can be inferred that common method bias did not present a significant problem in this study.

## 5. Results

### 5.1. Measurement Model

This study conducted confirmatory factor analysis (CFA) to test convergent validity and discriminant validity. In addition to the analysis of the model hypothesized in this study, confirmatory factor analysis was also conducted on four alternative models. As shown in Table 2, the hypothesized five-factor model (ESMU, SMUNW, SME, Resilience, TI) fit the data well (*χ*^2^*/df* = 2.882, CFI = 0.97, TLI = 0.96, RMSEA = 0.07, SRMR = 0.09) and was significantly better than other alternative models. Although some studies have set a more stringent standard for SRMR of no more than 0.08, many studies have proven that an SRMR of less than 0.1 is acceptable (Hu & Bentler, 1998, 1999), and the SRMR value of 0.9 in this study meets this standard. The composite reliability (CR) scores all exceeded 0.7 (ranging from 0.82 to 0.97), indicating that the convergent validity of the variable is good, and the AVE square root value of the variable is greater than the correlation coefficient value between it and other variables, confirming the verification of the discriminant validity of the variable. These results indicate that the variables involved in this study have good convergent and discriminant validity.

### 5.2. Descriptive Statistics and Correlations

The means, standard deviations, and correlations between variables in this study are shown in Table 3. Resilience is positively related to age (*r* = 0.15, *p* < 0.01), tenure (*r* = 0.14, *p* < 0.01), and marital status (*r* = 0.17, *p* < 0.001). ESMU is positively related to social media exhaustion (*r* = 0.57, *p* < 0.001) and turnover intention (*r* = 0.44, *p* < 0.001). SMUNW is positively related to social media exhaustion (*r* = 0.22, *p* < 0.001) and turnover intention (*r* = 0.15, *p* < 0.01). Social media exhaustion is positively related to turnover intention (*r* = 0.49, *p* < 0.001). These relationships are consistent with the hypothesized directions, and we proceeded with testing the hypotheses.

### 5.3. Hypothesis Testing

Hierarchical regression analysis was used to test the main effect and mediation effect, and the results are shown in Table 4. M3 showed that there was a positive relationship between ESMU and turnover intention (*β* = 0.42, *p* < 0.001); thus, H1a was supported. On the basis of M3, in M4, we added the mediating variable of social media exhaustion. The results showed that there was still a significant positive relationship between ESMU and turnover intention (*β* = 0.21, *p* < 0.001), which indicates that social media exhaustion plays a partial mediating role in the relationship between ESMU and turnover intention. In addition, the bootstrap analysis results indicated that the mediating effect of social media exhaustion between ESMU and turnover intention was 0.261, and the bootstrap confidence interval did not include 0 (95% CI = [0.171, 0.360]); thus, H2a was supported. M5 showed that there was a positive relationship between SMUNW and turnover intention (*β* = 0.14, *p* < 0.01); thus, H1b was supported. On the basis of M5, the mediating variable of social media exhaustion was added to M6. The results indicated that the relationship between SMUNW and turnover intention was no longer significant (*β* = 0.04, *p* > 0.05), which indicates that social media exhaustion plays a complete mediating role in the relationship between SMUNW and turnover intention. Furthermore, the bootstrap analysis results showed that the mediating effect of social media exhaustion between SMUNW and turnover intention was 0.160, and the bootstrap confidence interval excluded 0 (95% CI = [0.082, 0.246]); thus, H2b was supported.

This study explores the impact of the interaction of resilience with ESMU and SMUNW on social media exhaustion by constructing product terms, and the results are shown in Table 5. M8 revealed that the interaction term of resilience and ESMU showed a significant negative relationship with social media exhaustion (*β* = −0.11, *p* < 0.01); thus, H3a was supported. The results of M9 showed that the interaction term of resilience and SMUNW was significantly negatively related to social media exhaustion (*β* = −0.21, *p* < 0.001); thus, H3b was supported.

**Figure 2:**
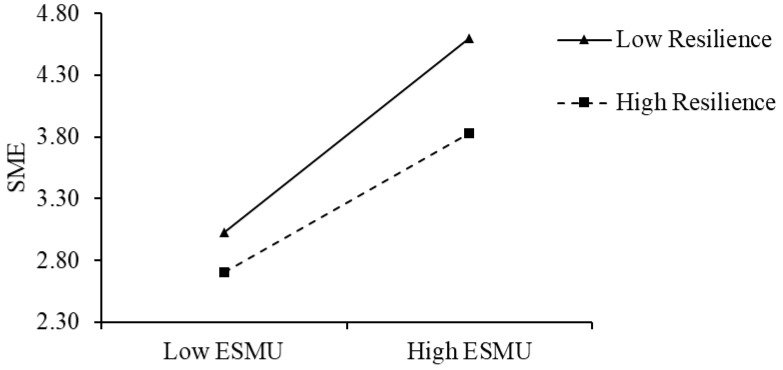
Interactive effect of ESMU and resilience on SME. N = 453. ESMU, excessive social media use at work. SME, social media exhaustion.

**Figure 3:**
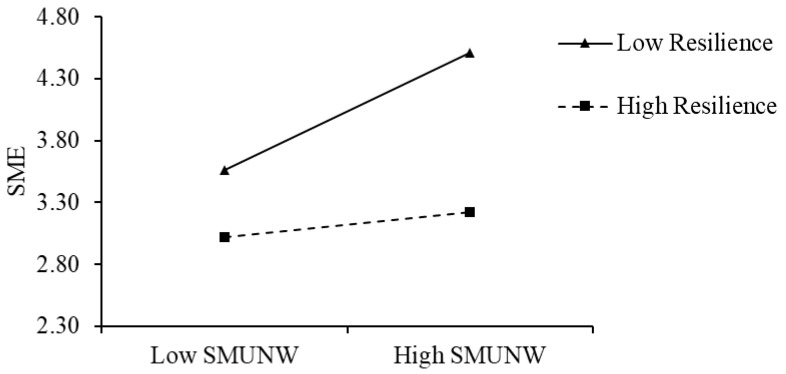
Interactive effect of SMUNW and resilience on SME. N = 453. SMUNW, social media use for work during non-work hours. SME, social media exhaustion.

In order to more clearly and intuitively show the magnitude and direction of the moderating effect of resilience on the above two relationships, in this study, we drew two simple slope diagrams to depict the relationship between ESMU and SMUNW and social media exhaustion under high (Mean + SD) and low (Mean − SD) resilience values. As shown in Figure 2, when resilience is higher, the positive effect of ESMU on social media exhaustion is weaker (simple slope = 0.50, *p* < 0.001) than when resilience is lower (simple slope = 0.70, *p* < 0.001). As shown in Figure 3, when resilience is higher, the positive impact of SMUNW on social media exhaustion is weaker (simple slope = 0.11, *p* > 0.05) than when resilience is lower (simple slope = 0.52, *p* < 0.001).

In order to test the moderated mediating effect, that is, to test the indirect effect of social media exhaustion between ESMU and SMUNW and turnover intention under different resilience levels, this study used the PROCESS MARCO developed by Hayes (2017), and the results are shown in Table 6. For the indirect effect of ESMU on turnover intention through social media exhaustion, at the lower level of resilience, the indirect effect is 0.278, and the bootstrap confidence interval for the indirect effect does not include zero (95% CI = [0.184, 0.379]). At the higher level of resilience, the indirect effect is 0.199, and the bootstrap confidence interval for the indirect effect does not include zero (95% CI = [0.123, 0.294]). The index of the moderated mediation is significant (index = −0.053), and the bootstrap confidence interval does not include zero (95% CI = [−0.095, −0.006]); thus, H4a is supported. For the indirect effect of SMUNW on turnover intention through social media exhaustion, at the lower level of resilience, the indirect effect is 0.274, and the bootstrap confidence interval for the indirect effect does not include zero (95% CI = [0.182, 0.365]). At the higher level of resilience, the indirect effect is 0.058, and the bootstrap confidence interval for the indirect effect includes zero (95% CI = [−0.041, 0.149]). The index of the moderated mediation is significant (index = −0.145), and the bootstrap confidence interval does not include zero (95% CI = [−0.215, −0.073]); thus, H4b is supported.

## 6. Discussion

The rapid development of ICT has accelerated the widespread adoption of social media for work-related purposes, both within and beyond formal working hours (Liu & Yuan, 2015). Despite the advantages of this type of use, the negative implications are the subject of growing academic and practical scrutiny (Blackwell et al., 2017; Hawi & Samaha, 2017; Su et al., 2020). Existing studies have predominantly examined excessive use within the workplace (Cao & Yu, 2019; B. Wang et al., 2023; Yu et al., 2018), whereas the emotional and behavioral consequences of non-work-related use remain underexplored. Furthermore, prior research has largely focused on private sector employees (Ali-Hassan et al., 2015; Kalra et al., 2023; Kanwal et al., 2023; Ma et al., 2023), resulting in a limited understanding of the situation among public sector employees. This limitation is particularly salient, as excessive social media use among civil servants has become increasingly prevalent in the context of digital governance (Sharif et al., 2015).

This study examined the adverse effects of ESMU and SMUNW on civil servants through social media exhaustion and the mitigating role of resilience. The findings aligned with our hypotheses based on COR theory. Both ESMU and SMUNW significantly increased social media exhaustion, which in turn fostered negative emotional states and behavioral tendencies. Consistent with COR theory, continuous social media demands erode psychological resources more strongly than resource acquisition (Hobfoll et al., 2018). This depletion arises from mechanisms such as information overload, interrupted recovery, and emotional regulation during professional social media use, which collectively accelerate exhaustion.

The results also showed that resilience moderated the relationship between social media exhaustion and its adverse outcomes, buffering the negative impact of digital demands. This extends COR theory to the public sector by underscoring the importance of personal resources in mitigating depletion processes (Halbesleben & Bowler, 2007). In the specific context of civil servants, resilience may be especially critical, as institutional expectations of constant responsiveness leave fewer opportunities for recovery compared with private sector employees. Accordingly, resilience constitutes not only a supplementary resource but also a necessary condition for sustaining well-being in digital governance.

Compared with prior research in private sector contexts (Ali-Hassan et al., 2015; Ma et al., 2023), our findings reveal both similarities and differences. Similarly to private employees, civil servants experience resource depletion and diminished well-being due to excessive and after-hours social media use. However, in contrast with private employees—whose outcomes are typically conceptualized in terms of reduced performance, engagement, or productivity—civil servants are subject to stricter institutional requirements, including accountability, administrative transparency, and continuous availability. These sector-specific demands intensify the adverse effects of SMUNW, rendering public employees particularly susceptible to exhaustion and turnover intention. Thus, while depletion mechanisms are generalizable, their manifestations are context-dependent, reflecting the distinctive pressures of public service.

These findings also resonate with those of international studies that document the detrimental effects of intensive social media use across cultural and organizational contexts (e.g., Blackwell et al., 2017; Hawi & Samaha, 2017; Su et al., 2020). By corroborating similar mechanisms in the Chinese public sector, this study reinforces the external validity of previous evidence while foregrounding sectoral and cultural contingencies. In particular, the Chinese context—characterized by strong digital governance, rapid ICT adoption, and high civil servant turnover—illustrates how structural and cultural factors exacerbate the costs of social media use. This underscores both the urgency and the relevance of addressing technostress in public administration.

Considered in their entirety, our findings enhance our understanding of how social media use influences civil servants’ psychological states and behaviors. Excessive and after-hours use depletes resources and leads to exhaustion, while resilience buffers these processes. By incorporating cross-sectoral comparisons and situating the analysis within the occupational context of Chinese civil servants, this study provides a nuanced account of technology-driven resource depletion and protection in the public sector.

### 6.1. Theoretical Implications

The theorical contributions of this study can be understood through five aspects. Firstly, this study included a comprehensive analysis of the use of social media during work and non-work hours. Although some scholars have focused on the adverse effects of the excessive use of social media (Blackwell et al., 2017; Hawi & Samaha, 2017; Su et al., 2020), most of these studies have focused on excessive social media use at work (ESMU), and little attention has been given to social media use for work during non-work hours (SMUNW). Meanwhile, to the best of our knowledge, there was no study prior that incorporated both ESMU and SMUNW. Thus, in the current study, we incorporated ESMU and SMUNW into a unified research model to explore their relative impacts on social media exhaustion and turnover intention. This study provided a more comprehensive and systematic study of social media use from the perspective of technostress.

Secondly, this study further elucidated the effect mechanism between social media use and turnover intention. The mediators between social media use and turnover intention in the existing literature mainly included work stress (Moqbel et al., 2020) and job engagement (X. Zhang et al., 2019), and there was limited research on the relationship between social media use and turnover intention from the perspective of exhaustion. Using social media exhaustion as a domain-specific type of exhaustion related to the use of social media for work purposes, this study posited and validated the mediating role of social media exhaustion between both types of social media use and their influence on turnover intention. Furthermore, we also added to the ongoing research on the antecedents of turnover intention in the public sector from the perspective of social media use and related domain-specific exhaustion. Future research should further explore the antecedents of social media exhaustion in order to work toward tackling it, as well as addressing related turnover intention, given that social media use is widely accepted and impossible to prevent due to the ubiquitousness of ICT in today’s work environment.

Thirdly, this study expanded the research on the boundary conditions of ESMU and SMUNW from the perspective of positive psychology. According to COR theory, when individuals face resource losses, they will use resource investment to cope with the situation (Halbesleben & Bowler, 2007). Different from resources gained from supervisors and the organization, resilience is a resource possessed by individuals and thus may be more convenient to refer to. In addition, resilience is also a positive psychological resource that is very helpful for individuals in difficult situations (Garcia-Dia et al., 2013). Thus, this study provided a method of mitigating the adverse influences of social media use from the perspective of individual resources.

Fourthly, this study diversified the scope of social media research. Social media use had previously mainly been examined in the context of private sector employees, with limited attention given to the public sector. However, the use of social media is not uncommon in the public sector (Sharif et al., 2015). With more and more public employees using social media extensively during working hours and also utilizing work social media in their own time, assessing social media-related technostress among them is both important and timely. Furthermore, public employees such as civil servants may be influenced by social media use even more because they may be on call 24/7. As such, this study is among the first attempts to examine social media use from the perspective of public personnel management, helping to raise awareness of the issue of the excessive use of social media in the public sector.

Finally, this study applied knowledge of positive psychology in the field of information and communication technology (ICT). Although scholars have conducted research on the negative effects of ICT (Karimikia et al., 2020; Scherer & Hatlevik, 2017), limited empirical efforts have been made to find moderators that can mitigate these impairments. Based on the resource investment principle of COR theory, this study viewed resilience as a positive psychological resource in the resource investment of civil servants when facing resource losses caused by ICT and empirically verified that resilience can attenuate the negative impacts of ICT. This study combined positive psychology with ICT and represents an important exploration of the integration of psychology into ICT research fields. Furthermore, our employment of COR theory in the context of social media use revalidated its utility in understanding the technostress phenomena.

### 6.2. Practical Implications

This study raises several practical implications in managing the risks associated with social media use among public employees.

Firstly, the results show that both excessive social media use at work (ESMU) and social media use during non-work hours (SMUNW) contribute to social media exhaustion and turnover intention. Managers should therefore avoid focusing exclusively on in-office digital behaviors and also recognize the hidden costs of after-hours use. Establishing clear boundaries for work-related social media activities and encouraging employees to disconnect outside working hours can reduce digital overload and protect their work–life balance.

Secondly, this study confirmed that resilience significantly mitigates the negative effects of ESMU and SMUNW. Public organizations should therefore recognize the importance of cultivating resilience at both the individual and organizational levels. In recruitment, resilience assessments could be incorporated to identify candidates with higher coping capacities. In employee development, training interventions such as stress inoculation training (SIT) (Vakili et al., 2014) and cognitive behavioral therapy (CBT) (Joyce et al., 2018) can be adopted to strengthen resilience as a psychological resource.

Thirdly, consistent with the resource investment principle of COR theory (Halbesleben & Bowler, 2007) and echoing prior empirical findings (Fang et al., 2024), the present study corroborates that resilience functions as a positive resource in the face of social media-related stress. This suggests that other forms of resource support—such as leadership care, supervisor support, and organizational programs aimed at improving psychological well-being—may also buffer the negative effects of social media use. Managers should therefore provide employees with a variety of supportive resources, ensuring that resilience development is embedded into a broader framework of organizational support.

Fourthly, our findings demonstrate that ESMU and SMUNW increase turnover intention through social media exhaustion, highlighting the importance of monitoring employees’ emotional exhaustion as a predictor of turnover intention (Saleh et al., 2023). Regular surveys or assessments of employees’ social media experiences can help managers to identify emerging stressors early and implement timely interventions to reduce the risk of attrition.

Finally, the results also have implications for policy design in the context of digital governance. Given that civil servants are often subject to institutional pressures regarding accountability and responsiveness, policy-makers should consider establishing guidelines that protect employees from excessive digital demands. For example, introducing “right to disconnect” policies, clarifying expectations for after-hours availability, and promoting digital well-being initiatives within the public sector can help create a healthier and more sustainable work environment (Weber & Llave, 2021). For these policies to be effectively operationalized in the Chinese public sector, managers could, for instance, clarify expectations for after-hours availability, set explicit cut-off times for digital communications, launch pilot “right to disconnect” programs in selected government agencies, and integrate digital well-being considerations into internal regulations, performance appraisal systems, and leadership accountability mechanisms. Nevertheless, cultural and institutional barriers—such as hierarchical traditions, strong expectations of constant responsiveness, and accountability pressures—may pose challenges in the enforcement of these policies. A gradual and context-sensitive approach, combining formal regulations with awareness campaigns and leadership role-modeling, may therefore be required to ensure that the “right to disconnect” becomes both legitimate and practicable in the Chinese context.

Together, these practical implications underscore the importance of managing social media use not only as an organizational or managerial issue but also as a broader matter of employee well-being and sustainable human resource management in the era of digital governance.

### 6.3. Limitations and Future Research Directions

The findings and contributions of the current study should be considered alongside its limitations. Firstly, this study adopted a cross-sectional research design, which is weak in the inference of causal relationships. Future work should use experimental research or longitudinal studies to further confirm the causal inferences. Secondly, this study collected self-reported data from the same source. Although we examined common method bias and the results indicated that common method bias was not a serious problem in this study, future work should use a multi-wave and multi-source research design to reduce common method bias. Thirdly, as this study was conducted with Chinese civil servants, the generalizability of the conclusions may be limited due to the influence of cultural and institutional specificities. The findings may have higher applicability to countries with administrative traditions and cultural contexts similar to those of China, whereas in substantially different settings, they should be interpreted with caution and subjected to further empirical validation.

## 7. Conclusions

This study found that both excessive social media use at work and social media use during non-work hours can deplete psychological resources, leading to social media exhaustion and, ultimately, turnover intention. Resilience was identified as a key personal resource that buffers these detrimental processes. These findings enrich the literature on technostress by revealing the mechanisms through which different forms of social media use affect public employees’ well-being and retention, while also extending the application of Conservation of Resources (COR) theory to the context of digital governance.

The implications of these findings are both theoretical and practical. Theoretically, this study integrates COR theory, technostress research, and positive psychology to provide a more comprehensive framework for understanding digital stressors in public personnel management. Practically, the results highlight the importance of public organizations recognizing the risks of excessive and after-hours social media use and actively supporting the development of resilience among civil servants. Policies that clarify digital work boundaries and training programs that develop coping capacities can help mitigate the negative impact of social media exhaustion.

Despite these contributions, this study is not without limitations. The use of cross-sectional and self-reported data limits causal inference and may raise concerns regarding common method bias, while the focus on civil servants in one province of China limits the generalizability. Future research could address these issues by using longitudinal or multi-source designs and testing the model across different sectors and cultural contexts.

In summary, this study demonstrates that digital technologies, while indispensable in modern governance, may also have significant negative effects on public employees. By highlighting both risks and protective factors, our study contributes to a more balanced understanding of how social media use shapes the work and well-being of civil servants in the digital era. Addressing technostress is therefore not only a matter of individual well-being but also a strategic necessity for sustaining employee retention and organizational effectiveness in the public sector. Recognizing these limitations, future research should extend this line of inquiry to different cultural and organizational settings to further validate and refine these insights.

## Figures and Tables

**Table 1:** Demographic characteristics of participants.

	Category	Frequency	Percent
Gender	Female	369	0.81
	Male	84	0.19
Age	21–29	160	0.35
	30–39	169	0.37
	40–49	70	0.16
	50–60	54	0.12
Tenure	1–5	136	0.30
	6–10	118	0.26
	11–15	66	0.15
	16–20	39	0.09
	21–30	52	0.11
	31–45	42	0.09
Marital status	Unmarried	116	0.26
	Married	337	0.74

N = 453.

**Table 2:** Confirmatory factor analysis results.

Model		*χ* ^2^	*df*	*χ* ^2^ */df*	CFI	TLI	RMSEA	SRMR
1. Hypothesized 5-factor model	ESMU, SMUNW, SME, Resilience, TI	420.80	146	2.88	0.97	0.96	0.07	0.09
2. Alternative 4-factor model	ESMU + SMUNW, SME, Resilience, TI	809.82	150	5.40	0.92	0.90	0.10	0.12
3. Alternative 3-factor model	ESMU + SMUNW, SME + Resilience, TI	1336.40	153	8.73	0.86	0.82	0.13	0.14
4. Alternative 2-factor model	ESMU + SMUNW + SME + Resilience, TI	2079.48	155	13.42	0.77	0.72	0.17	0.17
5. Alternative 1-factor model	ESMU + SMUNW + SME + Resilience + TI	3016.17	156	19.33	0.65	0.58	0.20	0.17

N = 453. ESMU, excessive social media use at work. SMUNW, social media use for work during non-work hours. SME, social media exhaustion. TI, turnover intention.

**Table 3:** Means, standard deviations, and correlations among variables.

	Mean	SD	1	2	3	4	5	6	7	8	9
1. Gender	0.19	0.39	-								
2. Age	35.08	9.26	0.21 ***	-							
3. Tenure	12.62	10.09	0.22 ***	0.94 ***	-						
4. Marital status	1.74	0.44	0.06	0.52 ***	0.48 ***	-					
5. ESMU	3.72	1.13	−0.02	0.03	0.02	−0.04	(0.89)				
6. SMUNW	4.45	0.92	−0.02	0.02	0.02	−0.06	0.51 ***	(0.83)			
7. SME	3.57	1.28	−0.01	0.04	0.02	0.03	0.57 ***	0.22 ***	(0.95)		
8. Resilience	4.22	0.75	0.09	0.15 **	0.14 **	0.17 ***	−0.26 ***	0.05	−0.32 ***	(0.68)	
9. TI	3.13	1.39	−0.05	−0.04	−0.03	−0.08	0.44 ***	0.15 **	0.49 ***	−0.42 ***	(0.92)

N = 453. The square roots of the AVE are the values in parentheses along the diagonal. ** *p* < 0.01, *** *p* < 0.001. ESMU, excessive social media use at work. SMUNW, social media use for work during non-work hours. SME, social media exhaustion. TI, turnover intention.

**Table 4:** Test results for main and mediation effects.

	SME			TI			
	M1	M2		M3	M4	M5	M6
Gender	0.00 (0.13)	−0.01 (0.16)		−0.03 (0.16)	−0.03 (0.15)	−0.04 (0.17)	−0.04 (0.15)
Age	0.11 (0.02)	0.18 (0.02)		−0.12 (0.02)	−0.17 (0.02)	−0.073(0.02)	−0.16 (0.02)
Tenure	−0.13 (0.02)	−0.17 (0.02)		0.12 (0.02)	0.17 (0.02)	0.09 (0.02)	0.17 (0.02)
Marital status	0.06 (0.13)	0.04 (0.16)		−0.09 (0.15)	−0.09 * (0.14)	−0.09 (0.17)	−0.10 (0.15)
ESMU	0.57 *** (0.04)			0.42 *** (0.05)	0.21 *** (0.06)		
SMUNW		0.22 *** (0.07)				0.14 ** (0.07)	0.04 (0.06)
SME					0.38 *** (0.05)		0.49 *** (0.05)
R^2^	0.33	0.05		0.19	0.29	0.03	0.26
∆R^2^	0.33	0.05		0.19	0.10	0.03	0.23
F	44.48 ***	4.90 ***		21.19 ***	30.52 ***	3.02 ***	26.38 ***

N = 453. ESMU, excessive social media use at work. SMUNW, social media use for work during non-work hours. SME, social media exhaustion. TI, turnover intention. Standardized coefficients are reported. Values in parentheses are standard error estimates. * *p* < 0.05, ** *p* < 0.01, *** *p* < 0.001.

**Table 5:** Test results for moderation effects.

	SME			
	M7	M8	M9	M10
Gender	0.01 (0.13)	0.02 (0.13)	0.01 (0.17)	0.03 (0.14)
Age	0.13 (0.02)	0.10 (0.02)	0.20 (0.02)	0.12 (0.02)
Tenure	−0.13 (0.01)	−0.10 (0.01)	−0.17 (0.02)	−0.10 (0.02)
Marital status	0.09 (0.13)	0.09 (0.13)	0.08 (0.15)	0.10 (0.15)
ESMU	0.52 *** (0.05)	0.53 *** (0.04)		
SMUNW			0.24 *** (0.06)	0.23 *** (0.05)
Resilience	−0.20 *** (0.05)	−0.21 *** (0.07)	−0.36 *** (0.06)	−0.36 *** (0.06)
ESMU ∗ Resilience		−0.11 ** (0.04)		
SMUNW ∗ Resilience				−0.21 *** (0.04)
R^2^	0.37	0.38	0.17	0.22
∆R^2^	0.37	0.01	0.17	0.04
F	43.57 ***	39.17 ***	15.61 ***	17.64 ***

N = 453. ESMU, excessive social media use at work. SMUNW, social media use for work during non-work hours. SME, social media exhaustion. TI, turnover intention. Standardized coefficients are reported. Values in parentheses are standard error estimates. ** *p* < 0.01, *** *p* < 0.001.

**Table 6:** Moderated mediation effect.

**ESMU——>SME——>Turnover Intention**				
**Resilience**	**Effect**	**BootSE**	**BootLLCI**	**BootULCI**
3.475 (M − SD)	0.278	0.050	0.184	0.379
4.222 (M)	0.238	0.044	0.159	0.330
4.970 (M + SD)	0.199	0.044	0.123	0.294
Moderated mediation	Index	BootSE	BootLLCI	BootULCI
	−0.053	0.023	−0.095	−0.006
SMUNW——>SME——>Turnover Intention				
Resilience	Effect	BootSE	BootLLCI	BootULCI
3.475 (M − SD)	0.274	0.047	0.182	0.365
4.222 (M)	0.166	0.039	0.088	0.241
4.970 (M + SD)	0.058	0.049	−0.041	0.149
Moderated mediation	Index	BootSE	BootLLCI	BootULCI
	−0.145	0.036	−0.215	−0.073

N = 453. ESMU, excessive social media use at work. SMUNW, social media use for work during non-work hours. SME, social media exhaustion. TI, turnover intention.

## Data Availability

The data presented in this study are available from the corresponding author on request.

## Appendix A

**Table A1:** Measurement items.

**Excessive social media use at work**
1. I think the amount of time l spend using social media at work is excessive.
2. I spend an unusually large amount of time using social media at work.
3. I spend more time using social media at work than most other people.
**Social media use for work during non-work hours**
1. I often use social media to connect with colleagues for work during non-work hours.
2. I felt obliged to respond to work-related messages from social media during non-work hours.
3. I often use social media to obtain work related information and knowledge during non-work hours.
4. I’m used to checking work related information on social media during non-work hours.
**Social media exhaustion**
1. I feel drained from activities that require me to use social media.
2. I feel tired from my social media activities.
3. Working all day with social media is a strain for me.
4. I feel burned out from my social media activities.
**Resilience**
1. When I have a setback at work, I have trouble recovering from it, moving on. (R)
2. I usually manage difficulties one way or another at work.
3. I can be “on my own,” so to speak, at work if I have to.
4. I usually take stressful things at work in stride.
5. I can get through difficult times at work because I’ve experienced difficulty before.
6. I feel I can handle many things at a time at this job.
**Turnover intention**
1. I often think of leaving this organization.
2. It is very possible that I will look for a new job next year.
3. Recently, I often think of changing the current job.
